# Investigation of body and udder skin surface temperature differentials as an early indicator of mastitis in Holstein Friesian crossbred cows using digital infrared thermography technique

**DOI:** 10.14202/vetworld.2016.1386-1391

**Published:** 2016-12-08

**Authors:** M. Sathiyabarathi, S. Jeyakumar, A. Manimaran, Heartwin A. Pushpadass, M. Sivaram, K. P. Ramesha, D. N. Das, Mukund A. Kataktalware, G. Jayaprakash, Tapas Kumar Patbandha

**Affiliations:** 1 Livestock Research Centre, Southern Regional Station, Indian Council of Agricultural Research - National Dairy Research Institute, Adugodi, Bengaluru - 560 030, Karnataka, India; 2 Dairy Engineering Section, Southern Regional Station, Indian Council of Agricultural Research - National Dairy Research Institute, Adugodi, Bengaluru - 560 030, Karnataka, India; 3 Dairy Economics and Statistics, Southern Regional Station, Indian Council of Agricultural Research - National Dairy Research Institute, Adugodi, Bengaluru - 560 030, Karnataka, India; 4 Dairy Production Section, Southern Regional Station, Indian Council of Agricultural Research - National Dairy Research Institute, Adugodi, Bengaluru - 560 030, Karnataka, India; 5 Department of Animal Nutrition, College of Veterinary and Animal Sciences, Mannuthy - 680 651, Kerala, India; 6 Livestock Production and Management Section, Indian Council of Agricultural Research - National Dairy Research Institute, Karnal - 132 001, Haryana, India

**Keywords:** diagnosis, lactating cows, mastitis, temperature

## Abstract

**Aim:**

The objective of this study was to investigate the ability of infrared thermography (IRT) technique and its interrelationship with conventional mastitis indicators for the early detection of mastitis in Holstein Friesian (HF) crossbred cows.

**Materials and Methods:**

A total of 76 quarters of lactating HF crossbred (*Bos indicus* × *Bos taurus*) cows (n=19) were monitored for body temperature (i.e., eye temperature) and udder skin surface temperature (USST) before milking using forward-looking infrared (FLIR) i5 camera. Milk samples were collected from each quarter and screened for mastitis using Somatic Cell Count (SCC), Electrical Conductivity (EC), and California mastitis test. Thermographic images were analyzed using FLIR Quick Report 1.2 image analysis software. Data on body and USST were compiled and analyzed statistically using SPSS 16.0 and Sigmaplot 11.

**Results:**

The mean±standard deviation (SD) body (37.23±0.08°C) and USST (37.22±0.04°C) of non-mastitic cow did not differ significantly; however, the mean USST of the mastitis-affected quarters were significantly higher than the body temperature and USST of unaffected quarters (p<0.001). The mean±SD USST of the subclinical mastitis (SCM) and clinical mastitis-affected quarters were 38.08±0.17 °C and 38.25±0.33 °C, respectively, which is 0.72 and 1.05 °C higher than the USST temperature of unaffected quarters. The USST was positively correlated with EC (r=0.95) and SCC (r=0.93). The receiver operating characteristic curve analysis revealed a higher sensitivity for USST in early prediction of SCM with a cut-off value of >37.61°C.

**Conclusion:**

It is concluded that infrared thermal imaging technique could be used as a potential noninvasive, quick cow-side diagnostic technique for screening and early detection of SCM and clinical mastitis in crossbred cows.

## Introduction

India ranks first in milk production, accounting for 18.5% of world milk production, achieving an annual output of 146.3 million tons during 2014-15 as compared to 137.69 million tons during 2013-14 recording a growth of 6.26%. Whereas, the Food and Agriculture Organization [1] has reported that 3.1% increase in world milk production from 765 million tons in 2013 to 789 million tons in 2014. However, with the increase in milk production, there is increase in the incidence of production diseases especially mastitis [2].

The dairy industry is facing a great setback due to the high prevalence and incidence of mastitis in dairy cattle [3]. The prevalence of subclinical mastitis (SCM) and clinical mastitis among non-descript, Deoni, Jersey and Holstein Friesian (HF) cows were 40.8%, 36.1%, 47.8%, and 54.7% and 3.8%, 1.8%, 10.1% and 13.2%, respectively [4]. The incidence of mastitis in Karan Fries, Karan Swiss, Sahiwal, and Tharparkar cows was reported to be 36.90%, 38.46%, 33.98% and 33.44%, respectively. The incidence was highest in fourth parity and above and lowest in first parities [5]. The global estimated economic loss per year due to mastitis amounts to USD 35 billion and ₹6000 crores for Indian dairy industry in which SCM is responsible for approximately 70-80% (₹4365 crore) of economic losses [6]. Milk production losses due to SCM amounted to 272 kg per lactation of nontreated cows in Lithuanian University Farm [7].

Early detection of mastitis is most important to prevent the losses associated with decreased milk production, quality and make decisions for quick and effective treatment. Skin temperature reflects the state of tissue metabolism and blood circulation; abnormal thermal patterns can signify areas of superficial inflammation or circulatory impairments. Infrared thermography (IRT) is employed as a diagnostic tool and shown to be sensitive enough to detect changes in udder skin surface temperature (USST) of healthy and mastitis-affected quarters [8-11]. A perusal of literature revealed that there are no reports available to establish whether a natural infection with a common course of events and its associated changes with mastitis are possible for detection by IRT in an organized dairy herd. This study was undertaken to assess the body and USST differentials using IRT for early detection of naturally occurring SCM and clinical mastitis in HF crossbred cows reared under subtropical conditions.

## Materials and Methods

### Ethical approval

Thermal imaging and milk sampling were performed as per the guidelines of the National Dairy Research Institute (NDRI) Animal Ethical Committee for care and use of experimental animals.

### Study area and experimental cows

The study was conducted at Livestock Research Centre (LRC), Southern Regional Station (SRS) of Indian Council of Agricultural Research - NDRI located at an altitude of 1200 m above sea level on 12°58’53″ N latitude and 77°36’42″ E longitudes. The climatic condition of the farm is of subtropical where temperature raises up to 36 °C in summer and comes closer to 15 °C during the winter season. The average rainfall ranges from 800 to 1200 mm and the maximum is received during July to October.

In this study, a total of 76 quarters of apparently healthy lactating HF crossbred (*Bos indicus* × *Bos taurus*) cows (n=19) from first to sixth parity with an average body weight of 451±19 kg and milk yield of 14.4±0.2 kg per cow were used. In this study, mastitis was not induced experimentally in any cows, and IRT imaging was done daily in all the cows to detect naturally occurring mastitis due to infection/environmental factors. The animals were maintained under loose housing system and milked twice daily with machine milking system. The cows were provided with recommended concentrate feed, fodder (green and dry) and had free access to water.

### Thermal imaging and milk sampling

A total number of 1064 eyes and 4256 skin surface thermal images of udder quarter were taken before morning and evening milking using forward-looking infrared (FLIR) i5 camera (FLIR Systems, Inc. 27700 SW Parkway Ave. Wilsonville, OR 97070, USA) continuously for 28 days. Before capturing the image, the camera was calibrated to ambient temperature, and the temperature measurement was adjusted to degree Celsius and distance to meters. The value of emissivity and reflected apparent temperature was kept constant for all the images as 0.98 and 20.0°C, respectively. A lateral thermographic image of the eye was taken at a distance of 1.0 m from the lateral side of animal’s head to observe cow’s body temperature. Thermographic images of udder were taken before milking at a distance of 1.0 m from the udder. Thermographic images were captured from the lateral side for forequarters and posterior or lateral side for hind quarters of the udder. The thermographic images were analyzed by FLIR Quick Report 1.2 software. The temperature of the inner canthus and the maximum temperature of the udder surface in a particular image was recorded and used in the analysis.

After capturing thermal images of all the quarters, milk samples from each quarter were collected separately during morning milking in a clean polystyrene tube and tested immediately to Somatic Cell Count (SCC) (The PortaSCC^®^ Somatic Cell Test, Whittendale Drive, Suite E Moorestown, NJ 08057 USA), Electrical Conductivity (EC) (Draminski Electronic Mastitis Detector, DRAMINSKI Ul. Owocowa 17 10-860 Olsztyn Poland) and California Mastitis Test (CMT) (Immucell Corporation, 56 Evergreen Drive, Portland) using standard protocol. The quarter which showed SCC of more than 4 lakhs cells per ml of milk in a crossbred cow was considered as mastitis-affected animals [12]. Besides, the animals with CMT score of trace and more than one and cows with more than 50-unit difference of EC values in between four quarters were also considered as mastitis-affected animals.

### Statistical analysis

An analysis of variance was employed to compare the non-mastitis, SCM and clinical mastitis-affected cows with respect to body temperature, USST, EC, and SCC. The correlation between the USST and mastitis indicators of non-mastitis and mastitis-affected cows were performed using a Pearson’s test. Correlation <0.3 were considered weak, between 0.3 and 0.7 were moderate and above 0.7 were strong. The regression model was employed relating USST with mastitis indicators of non-mastitis and mastitis-affected cows. Receiver operating characteristic (ROC) curve was plotted to compare the sensitivity and specificity of IRT with another test to detect SCM. All the above statistical analysis was made using SPSS 16.0 (IBM Corporation, Armonk, New York, USA) and Sigma plot 11 (SYSTAT, Salano, California, USA).

## Results and Discussion

Mastitis is the most common and important economic disease of the dairy industry and has a significant effect on quality of milk [13] and udder health. Early detection of mastitis is important for effective and successful treatment of intramammary infection. There are several techniques and biomarkers are available for early detection of changes in milk associated with SCM and clinical mastitis. However, these diagnostic methods are laboratory oriented, lack full accuracy and needs a considerable time of farm staff or milker [8,9,11]. Therefore, a cost-effective, rapid, non-invasive cow-side diagnostic technique with potential application in the field is essential for monitoring udder health.

**Figure-1:**
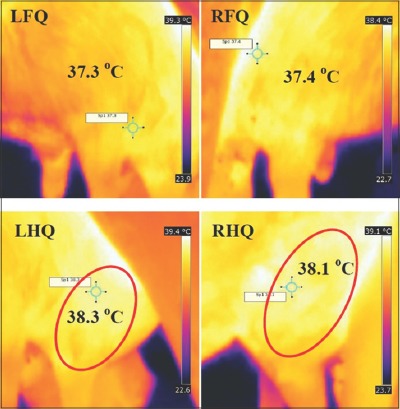
Infrared thermography thermogram of subclinical mastitis-affected Holstein Friesian crossbred cow from the lateral side of udder quarters. LFQ: Left fore quarter, RFQ: Right fore quarter, LHQ: Left hind quarter, RHQ: Right hind quarter. LHQ and RHQ showing increased temperature.

Automated methods for early and reliable detection of mastitis are currently in focus under precision dairying. In this study, IRT was found as a rapid and non–invasive cow-side technique for early detection of SCM in crossbred cows. Several studies demonstrated the use of IRT for early detection of SCM and clinical mastitis in dairy cows and ewes [8,11,14]. Skin temperature reflects the status of tissue metabolism, blood circulation, and abnormal thermal patterns can signify areas of superficial inflammation or circulatory impairments. Clinically, inflammation is characterized by five cardinal signs namely rubor (redness), calor (increased heat), tumor (swelling), dolar (pain), and functio laesa (loss of function). The inflammatory response in mastitis is initially associated with the rise in the temperature of the udder. IRT detects surface heat emitted as infrared radiation. A thermal camera absorbs infrared radiation and generates pictorial images based on the amount of heat generated, without causing radiation exposure [15]. In general, on a thermograph, the warmest areas appear white or red whereas the coolest regions appear blue or black (Figure-1) [10].

**Figure-2:**
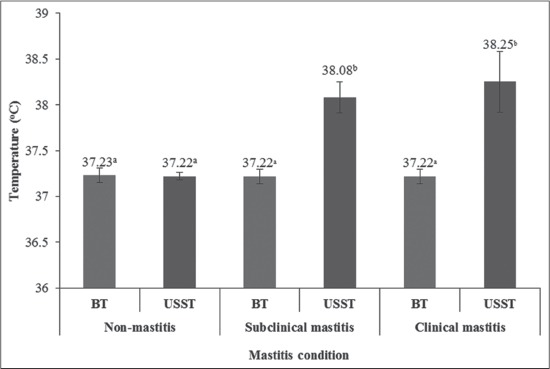
Temperature difference between body and udder skin surface of non-mastitis and mastitis affected crossbred cows. BT: Body temperature, USST: Udder skin surface temperature.

In this study, the USST (mean±standard deviation [SD]/range) of the SCM (38.08±0.17°C/37.90-38.4°C) and clinical mastitis-affected quarter (38.25±0.33°C/37.90-38.90°C) of HF crossbred cows was higher than temperature of body (37.23±0.08°C/37.15-37.31°C) and non-mastitis quarter (37.22±0.05°C/37.14-38.4°C) by 0.86-1.02°C (Figure-2). Hovinen *et al*. [9] who observed an increase of 1-1.5°C in USST associated with lipopolysaccharide (LPS) induced clinical mastitis using IRT in Ayrshire and HF cows. Hurnik *et al*. [16] suggested that monitoring udder health for detection of mastitis revealed that USST started increasing 3 days before clinical changes and they could detect four out of six cases earlier using IRT and detection ability increased in severe cases. Temperature increases of +2.3°C of USST were detectable through IRT in experimentally induced mastitis by infusion of bacterial endotoxin [17]. Similarly, Hovinen *et al*. [9] demonstrated that IRT was capable of detecting 1-1.5°C change in temperature difference in udder skin surface associated with clinical mastitis induced by *Escherichia coli* endotoxin. The thermal camera detects increased USST both in experimentally induced [9,17] and in natural course of infection as demonstrated in this study where the increased USST could be the result of vascular dilatation, loss of capillary permeability and hyperthermia at the site of infection [18].

In this study, affected quarters showed a higher temperature than the unaffected quarters and the temperature differential was 1.0°C. Willits [19] observed that mastitis causes USST to rise often before other clinical signs are visible. Scott *et al*. [17] detected a clear rise in temperature of the experimentally induced quarters than the control quarters. Similarly, Hovinen *et al*. [9] observed a transient rise in rectal temperature of experimentally induced clinical mastitis with the simultaneous rise of USST, which was successful, detected using the thermal camera. However, using IRT they could not detect local inflammatory changes of the udder, appearing earlier than the rectal temperature increase. Metzner *et al*. [11] demonstrated a good correlation between USST and rectal temperature in relation to mastitis-affected hind quarters.

Interestingly, Barth [20] observed that the mean surface temperature of teats increased from 30.1°C at the tip to 35.1°C at the udder base and USST was higher for mastitis-affected quarters (34.1°C) with SCC of >100,000 than unaffected (or) healthy quarter (33.6°C) with SCC of <100,000, and the temperature differential between affected and healthy quarter was 0.5°C.

Certain cells especially leukocytes presence and its quantity level in the milk are directly related to the severity of infection and type of causative pathogen [21]. SCM is characterized by apparently normal milk but with an increase in SCC of up to 400,000 cells per ml [22] which further reflects the severity of the infection. Diagnosis of SCM can be made through many methods including direct measurement of SCC or indirectly by performing CMT on suspected quarters. In most of the time, CMT is employed as an appropriate cow-side test for evaluation of udder health reflects the SCC level quite accurately and is a reliable indicator of the severity of infection [23].

Detection of clinical mastitis is mainly based on abnormal milk with SCC of >500,000 per ml and inflammatory changes viz. swelling, pain and consistency, which also depends on the severity of infection and causative organism [24]. Besides, increased SCC and CMT, the score was associated with isolation of causative organism on the microbiological culture of milk sample [25,26]. Alterations in EC are of critical importance to assess the quality of milk. The EC of normal milk from healthy quarters varies between 4 and 5.5 mS/cm at 25°C [27] and differential values of EC between four quarters are compared to find out abnormal values related to mastitis-affected quarters. During mastitis, the milk has a higher EC value than normal milk of unaffected quarter, which is due to udder tissue damage and subsequent increase in sodium (Na^+^) and chloride (Cl^−^) ions in milk [23]. Therefore, changes in EC of milk, when measured in tandem with SCC or CMT score and USST, would serve as a valid diagnostic methodology for detecting early onset of mastitis. Hovinen *et al*. (2008) observed that LPS challenged mastitis quarter showed increased USST 4-h post challenge than control quarters by 0.6°C, and this temperature rise was significantly (p<0.001), parallel with an increase in SCC and EC value of milk from challenged quarters.

**Figure-3:**
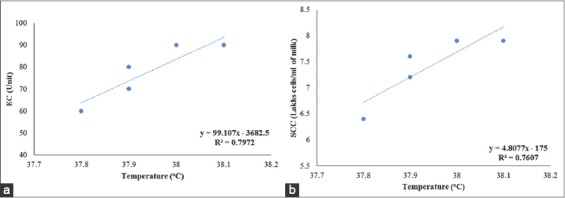
(a and b) Linear regression relating udder skin surface temperature with electrical conductivity (EC) and somatic cell count (SCC) of subclinical mastitis-affected crossbred cows.

Polat *et al*. [8] studied the interrelationships between USST and other mastitis indicators, viz., SCC and CMT score. The USST was positively correlated with SCC and the CMT score (r=0.86; p<0.0001), SCM quarter with SCC >400,000 cells per ml showed UST 2.35°C more than the healthy quarter with SCC of <400,000 cells per ml, which is similar to the present results of our study where a moderate correlation of USST with SCC was observed. The mean±SD SCC values (lakhs cells per ml of milk) were 2.05±0.85 (0.6-3.6), 8.16±0.99 (6.4-9.6) and 21.05±1.34 (20.1-22.0) and the mean USST values (°C) were 37.22±0.04, 38.08±0.18 and 38.25±0.33 for the quarters with CMT score negative (n=57), trace (n=11) and +1 (n=8), respectively (Table-1 & 2). The mastitis indicators studied in this study were interrelated. The USST was positively correlated with EC and SCC (0.95 and 0.93). This relationship was, expectedly linear as the EC and SCC values increased, USST linearly increased (0.79 and 0.76) (Figure-3). In this study, out of 19 animals only one animal which was detected for SCM by IRT method, showed clinical mastitis in due course of time.

**Table-1:** Descriptive statistics of the USST (°C); EC (unit); and SCC (lakhs) by CMT score.

CMT score									

Descriptive measures	Negative (n=57)			Trace (n=11)			One and above (n=8)		
		
	USST (°C)	EC (Unit)	SCC (Lakhs)	USST (°C)	EC (Unit)	SCC (Lakhs)	USST (°C)	EC (Unit)	SCC (Lakhs)
Mean	37.22	12.00	2.05	38.08	66.36	8.16	38.25	146.25	21.05
SE	0.01	1.56	0.19	0.05	6.91	0.31	0.12	12.81	0.95
Median	37.21	10.00	2.10	38.10	60.00	7.95	38.15	145.00	21.05
SD	0.04	6.96	0.85	0.17	22.92	0.99	0.33	36.23	1.34
Range	0.17	30.00	3.00	0.50	90.00	3.20	1.00	100.00	1.90
Minimum	37.14	0.00	0.60	37.90	10.00	6.40	37.90	100.00	20.10
Maximum	37.31	30.00	3.60	38.40	100.00	9.60	38.90	200.00	22.00

USST=Udder skin surface temperature, EC=Electrical conductivity, SCC=Somatic cell count, CMT=California mastitis test, SE=Standard error, SD=Standard deviation

**Table-2:** Comparison of body temperature, USST, EC and SCC (mean±SD) of non-mastitis and mastitis-affected HF crossbred cows.

Condition	BT (°C)	USST (°C)	EC (Unit)	SCC (Lakhs/ml)
Non-mastitis	37.23±0.08	37.22±0.04^a^	12.0±6.95^a^	2.04±0.85^a^
SCM	37.22±0.04	38.08±0.17^b^	58.18±25.65^b^	8.16±0.99^b^
Clinical mastitis	37.22±0.08	38.25±0.33^c^	146.25±36.22^c^	21.05±1.34^c^
p value	0.712	0.000	0.000	0.000

Values with different superscripts with in the column differ significantly at p<0.001, BT=Body temperature, USST=Udder skin surface temperature, EC=Electrical conductivity, SCC=Somatic cell count, HF=Holstein Friesian, SD=Standard deviation

**Figure-4:**
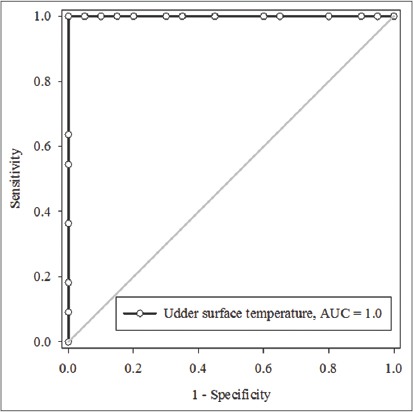
Area of udder skin surface temperature under the receiver operating characteristic curve.

The ROC curve analysis revealed a higher sensitivity and specificity at 95% confidence interval with a range of 71.51-100% and 83.16-100%, respectively, for USST with SCC and CMT in early prediction of SCM quarter (>4 lakhs/ml) with a cut-off value of >37.61°C in crossbred cows. The USST of >37.61°C was set as cut-off value in early prediction of SCM (Figure-4). This finding agrees with Polat *et al*. [8] they found the USST (mean±standard error) of 33.45±0.09°C and 35.80±0.08°C for healthy (<4 lakhs cells/ml) and SCM (>4000 lakhs/ml) affected quarters. The sensitivity and specificity were 95.6 and 93.6 for IRT and 88.9 and 98.9 for CMT in relation to SCM.

## Conclusion

This study demonstrates for the first time that in HF crossbred cows the body and USST differentials with a cut-off value of >37.61°C could detect the early occurrence of SCM in a dairy herd when monitored continuously using IRT. The results of the present study in lactating HF crossbred cows suggest that IRT was sensitive enough to detect thermal changes and rise in USST in relation to naturally occurring SCM and clinical mastitis. The diagnostic reliability is also well correlated with other mastitis indicators, viz., SCC, CMT and EC, indicating IRT of udder could be employed as one of the most potential, non-invasive, quick, cow-side diagnostic tool to monitor udder health and early detection of SCM in dairy animals.

## Authors’ Contributions

MS, SJ, AM, HAP, MS, KPR, DND, and MAK have formulated the research plan, MS, SJ and AM conducted experimental procedures and prepared the drafted manuscript. GJ and TKP assisted in collecting and compiling the resource material and in manuscript preparation. All authors read and approved the final manuscript.

## References

[1] Food and Agricultural Organization (FAO) (2014). Food Outlook Biannual Report on Global Food Market.

[2] Sharma N, Rho G.J, Hong Y.H, Kang T.Y, Lee H.K, Hur T.Y, Jeong D.K (2012). Bovine mastitis:An Asian perspective. Asian J. Anim. Vet. Adv.

[3] Halasa T, Huijps K, Osteras O, Hogeveen H (2007). Economic effects of bovine mastitis and mastitis management:A review. Vet. Q.

[4] Kurjogi M.M, Kaliwal B.B (2014). Epidemiology of bovine mastitis in cows of Dharwad District. Int. Sch. Res. Notices.

[5] Jingar S.C, Mehla R.K, Singh M, Kumar A, Kantwa S.C, Singh N (2014). Comparative study on the incidence of mastitis during different parities in cows and buffaloes. Indian J. Anim. Res.

[6] Antanaitis R, Zilaitis V, Juozaitiene V, Palubinskas G, Kucinskas A, Sederevicius A, Beliavska-Aleksiejune D (2015). Efficient diagnostics and treatment of bovine mastitis according to herd management parameters. Vet. Med. Zootec.

[7] Dua K (2001). Incidence, etiology and estimated economic losses due to mastitis in Punjab and in India-an update. Indian Dairyman.

[8] Polat B, Colak A, Cengiz M, Yanmaz L.E, Oral H, Bastan A, Kaya S, Hayirli A (2010). Sensitivity and specificity of infrared thermography in detection of subclinical mastitis in dairy cows. J. Dairy Sci.

[9] Hovinen M, Siivonen J, Taponen S, Hanninen L, Pastell M, Aisla A.M, Pyorala S (2008). Detection of clinical mastitis with the help of a thermal camera. J. Dairy Sci.

[10] Colak A, Polat B, Okumus Z, Kaya M, Yanmaz L.E, Hayirli A (2008). Short communication:Early detection of mastitis using infrared thermography in dairy cows. J. Dairy Sci.

[11] Metzner M, Sauter-Louis C, Seemueller A, Petzl W, Klee W (2014). Infrared thermography of the udder surface of dairy cattle:Characteristics, methods, and correlation with rectal temperature. Vet. J.

[12] Saravanan R, Das D.N, De S, Panneerselvam S (2015). Effect of season and parity on somatic cell count across zebu and crossbred cattle population. Indian J. Anim. Res.

[13] Bar D, Tauer L.W, Bennett G, Gonzalez R.N, Hertl J.A, Schukken Y.H, Schulte H.F, Welcome F.L, Grohn Y.T (2008). The cost of generic clinical mastitis in dairy cows as estimated by using dynamic programming. J. Dairy Sci.

[14] Costa A.C, Caja G, Salama A.A.K, Rovai M, Flores C, Aguilo J (2014). Thermographic variation of the udder of dairy ewes in early lactation and following an *Escherichia coli* endotoxin intramammary challenge in late lactation. J. Dairy Sci.

[15] Kunc P, Knizkova I, Prikryl M, Maloun J (2007). Infrared thermography as a tool to study the milking process. Agric. Trop. Subtrop.

[16] Hurnik J.F, De Boer S, Webster A.B (1984). Detection of health disorders in dairy cattle utilizing a thermal infrared scanning technique. Can. J. Anim. Sci.

[17] Scott S.L, Schaefer A.L, Tong A.K.W, Lacasse P (2000). Use of infrared thermography for early detection of mastitis in in dairy cows. Can. J. Anim. Sci.

[18] Jones B.F, Plassmann P (2002). Digital infrared thermal imaging of human skin. IEEE Eng. Med. Biol.

[19] Willits S (2005). Infrared Thermography for Screening and Early Detection of Mastitis in Working Dairy Herds. InfraMation 2005 Proceedings.

[20] Barth K (2000). Basic investigations to evaluate a highly sensitive infrared-thermograph technique to detect udder inflammation in cows. Milk Sci. Int.

[21] Viguier C, Arora S, Gilmartin N, Welbeck K, O’kennedy R (2009). Mastitis detection:Current trends and future perspectives. Trends Biotechnol.

[22] Sears P.M, McCarthy K.K (2003). Diagnosis of mastitis fortherapy decisions. Vet. Clin. North Am. Food Anim. Pract.

[23] New York State Cattle Health Assurance Program Veterinary Resource Diagnosis of Mastitis and Diagnostic Method for Investigating Udder Health Problems.

[24] McDougall S (1998). Efficacy of two antibiotic treatments in curing clinical and subclinical mastitis in lactating dairy cows. N. Z. Vet. J.

[25] McDermott M.P, Erb H.N, Natzke R.P (1982). Predictability by somatic cell counts related to prevalence of intramammary infection within herds. J. Dairy Sci.

[26] Sargeant J.M, Leslie K.E, Shirley J.E, Pulkrabek B.J, Lim G.H (2001). Sensitivity and specificity of somatic cell count and California mastitis test for identifying intramammary infection in early lactation. J. Dairy Sci.

[27] Hillerton J.E, Walton A.W (1991). Identification of subclinical mastitis with hand held electrical conductivity meter. Vet. Res.

